# Engagement With a Mobile Phone–Based Life Skills Intervention for Adolescents and Its Association With Participant Characteristics and Outcomes: Tree-Based Analysis

**DOI:** 10.2196/28638

**Published:** 2022-01-19

**Authors:** Raquel Paz Castro, Severin Haug, Rudolf Debelak, Robert Jakob, Tobias Kowatsch, Michael P Schaub

**Affiliations:** 1 Swiss Research Institute for Public Health and Addiction University of Zurich Zurich Switzerland; 2 Department of Psychology, Psychological Methods, Evaluation and Statistics University of Zurich Zurich Switzerland; 3 Wilhelm Wundt Institute for Psychology University of Leipzig Leipzig Germany; 4 Centre for Digital Health Interventions, Department of Management, Technology, and Economics ETH Zurich Zurich Switzerland; 5 Centre for Digital Health Interventions Institute of Technology Management University of St.Gallen St.Gallen Switzerland

**Keywords:** engagement, life skills, adolescents, mobile phone, machine learning, decision tree, mobile phone

## Abstract

**Background:**

Mobile phone–delivered life skills programs are an emerging and promising way to promote mental health and prevent substance use among adolescents, but little is known about how adolescents actually use them.

**Objective:**

The aim of this study is to determine engagement with a mobile phone–based life skills program and its different components, as well as the associations of engagement with adolescent characteristics and intended substance use and mental health outcomes.

**Methods:**

We performed secondary data analysis on data from the intervention group (n=750) from a study that compared a mobile phone–based life skills intervention for adolescents recruited in secondary and upper secondary school classes with an assessment-only control group. Throughout the 6-month intervention, participants received 1 SMS text message prompt per week that introduced a life skills topic or encouraged participation in a quiz or individual life skills training or stimulated sharing messages with other program participants through a friendly contest. Decision trees were used to identify predictors of engagement (use and subjective experience). The stability of these decision trees was assessed using a resampling method and by graphical representation. Finally, associations between engagement and intended substance use and mental health outcomes were examined using logistic and linear regression analyses.

**Results:**

The adolescents took part in half of the 50 interactions (mean 23.6, SD 15.9) prompted by the program, with SMS text messages being the most used and contests being the least used components. Adolescents who did not drink in a problematic manner and attended an upper secondary school were the ones to use the program the most. Regarding associations between engagement and intended outcomes, adolescents who used the contests more frequently were more likely to be nonsmokers at follow-up than those who did not (odds ratio 0.86, 95% CI 0.76-0.98; *P*=.02). In addition, adolescents who read the SMS text messages more attentively were less likely to drink in a problematic manner at follow-up (odds ratio 0.43, 95% CI 1.29-3.41; *P*=.003). Finally, participants who used the program the most and least were more likely to increase their well-being from baseline to 6-month follow-up compared with those with average engagement (*βs*=.39; t_586_=2.66; *P*=.008; *R^2^*=0.24).

**Conclusions:**

Most of the adolescents participating in a digital life skills program that aimed to prevent substance use and promote mental health engaged with the intervention. However, measures to increase engagement in problem drinkers should be considered. Furthermore, efforts must be made to ensure that interventions are engaging and powerful across different educational levels. First results indicate that higher engagement with digital life skills programs could be associated with intended outcomes. Future studies should apply further measures to improve the reach of lower-engaged participants at follow-up to establish such associations with certainty.

## Introduction

### Background

Adolescence is a vulnerable period in life, during which substance use and mental disorders often first emerge and begin to develop [1]. In the age group of 11-15 years, the prevalence of lifetime and recent alcohol and tobacco use shows a sharp increase in both genders [2]. In Switzerland, for example, the lifetime prevalence of alcohol use increased from 22% among boys aged 11 years to 70% among boys aged 15 years and from 11% in girls aged 11 years to 69% in girls aged 15 years [3]. The lifetime prevalence of tobacco use increased from 6% in boys aged 11 years to 35% in boys aged 15 years and from 2% in girls aged 11 years to 30% in girls aged 15 years. Taxation, public consumption bans, advertising restrictions, and raising the minimum legal age have proven to be effective ways to prevent substance use during adolescence. However, early interventions that incorporate skills trainings have also shown promising effectiveness [4]. Evidence for this effectiveness primarily stems from life skills trainings embedded in school curricula [5-11], whereas life skills trainings to promote mental health and prevent substance use in young adolescents are only recently being adapted to digital interventions as well as being tested [12-18]. This trend is mainly fostered by the difficulties that schools encounter when trying to implement life skills trainings in their curricula [9] and the personnel and financial resources that such programs require [19]. Digital interventions have great potential to overcome these obstacles. Such interventions have a large reach at low cost and can deliver uniquely personalized content automatically, which can be accessed at any time and anywhere [4]. The main disadvantage of delivering life skills training electronically is the lack of control over adolescents’ engagement with the intervention. Several reviews on digital interventions to promote mental health [20-22] or to prevent substance use [23] in young people point at the relatively low levels of user engagement. However, the most common assumption in current literature is that some form of engagement is essential for digital interventions to be effective [24-26]. Engagement, for instance, has been conceptualized in previous studies as both “(1) the extent (e.g. amount, frequency, duration, depth) of usage and (2) a subjective experience characterised by attention, interest and affect” [25]. This definition will be used in the context of this paper. From studies on adult populations, there is some evidence on factors that may influence engagement with digital behavior change interventions [25]. The conceptual framework of Perski et al [25] specifies factors that might affect engagement, including characteristics of the intervention (eg, esthetics or design, ease of use, and the inclusion of known behavior change techniques such as feedback and goal setting) or the context (eg, norms, age, education, and self-efficacy). Other factors were hypothesized to influence engagement (eg, the target behavior itself and some mechanism of action, such as accountability and motivation). Most of the current knowledge on predictors of engagement in adolescent populations comes from digital interventions devoted to physical activity [27,28]. To date, only a few studies have examined predictors of engagement with digital interventions devoted to mental health or substance use in adolescents. Furthermore, all studies examined engagement in terms of program use and not in terms of a subjective experience. In an internet-based depression prevention program directed at young adolescents from Australian public schools, low engagement with the program was predicted by being older, living in an urban area, or having lower leves of depressive symptoms or self-esteem at baseline [27]. In a mobile phone–based smoking cessation intervention targeting Swiss adolescents from secondary schools, nonengagement over time was most common among older smokers, smokers with an immigrant background, and smokers who reported nonproblematic levels of alcohol use at baseline [29]. Because of the novelty of using digital life skills interventions to promote healthy lifestyles in adolescents, it is still unclear which characteristics predict user engagement. Ascertaining the predictors in this new field can reveal who engages the most and for whom new strategies to increase program engagement have to be developed.

Besides the question of *who engages*, there is a wider debate on the associations between engagement and outcomes. Adolescents’ engagement with digital interventions on mental health has been found to be positively [30], negatively [31], or not linked [27] to intended behavior changes. Furthermore, decreasing engagement with a digital intervention—but not stable engagement—over time was associated with a smoking reduction in adolescents compared with nonengagement [29]. Which association between engagement and outcomes is observed depends on the selected engagement measure (eg, number of log-ins vs completion of a specific module) and on the underlying motivation for disengagement (eg, loss of interest vs sufficient support) [24,26,32,33]. New approaches try to operationalize *effective engagement* with the intervention, defined as the degree of engagement needed to achieve intended outcomes [34]. Given the current lack of knowledge about digital life skills interventions, a comprehensive approach to the assessment of indicators of engagement is needed. Furthermore, a broad approach must be taken to assess predictors of engagement, including previous known and unknown characteristics possibly related to program use and subjective experience. As use data tend to be highly skewed [35], methods are needed that (1) can select predictors in a consistent and unbiased way and (2) overcome distributional assumptions made by standard methods such as regression analysis.

To sum up, studies on engagement need to address different methodological challenges, through (1) a clear definition of engagement, (2) a selection of appropriate engagement measures, and (3) appropriate statistical methods that can handle large amount of data with different distributional characteristics.

### Objective

This study aims to use decision trees to determine predictors of engagement within a randomized controlled trial assessing the effectiveness of a novel mobile phone–based life skills intervention for secondary and upper secondary school students. The intervention addressed (1) self-management skills, (2) social skills, and (3) substance use resistance skills, which are the central competences included in widely disseminated life skills programs [7,36,37]. Decision trees allow the selection of relevant predictors associated with the outcome of interest and display them in a way that is easy to interpret. Furthermore, the relationship between engagement and changes in outcomes will be analyzed.

## Methods

### Participants and Procedures

Data for this study were extracted from a 2-arm, parallel-group, cluster randomized controlled trial that used school class as the randomization unit, as detailed elsewhere [14,18].

Students in secondary and upper secondary schools in the German-speaking part of Switzerland were invited by cooperating regional centers for addiction prevention to participate in a mobile phone–based program called *SmartCoach* if they (1) were aged ≥14 years, (2) could provide informed parental consent (only necessary for those aged 14 years), and (3) owned a mobile phone. The participating students were reimbursed CHF 10 (US $10.90) for participating in the 6- and 18-month follow-up assessments. To increase program engagement, a friendly competition among the participants was integrated into the program. This form of friendly competition was encouraged by answering SMS text messages, creating messages or taking pictures within contests, or assessing video and website links integrated into SMS text messages. With every interaction, the participants were able to collect credits, and the more credits the participants collected, the higher their chances of winning 1 of 10 prizes, which were part of a prize draw (10×CHF 50 [US $54.50] in cash). The participants had constant access to their current credit score on the personal profile page, which also displayed scores of the other participants in the same group (same starting date).

In the original trial, the efficacy of the life skills intervention *SmartCoach* was tested against an assessment-only control group. A total of 1473 students (mean age 15.4, SD 1.0, years; 813/1473, 55.19%, were girls) from 89 secondary and upper secondary school classes in the German-speaking part of Switzerland participated in this study. They were randomly assigned to either the intervention (750/1473, 50.92%; 44 classes) or to the assessment-only control group (723/1473, 49.08%; 45 classes). In this study, only the participants assigned to the intervention group were examined. Of the 750 participants in the intervention group, 597 (79.6%) took part in the 6-month follow-up assessment. Attrition at 6-month follow-up was significantly associated with use of the program (t_423.14_=25.3; *P*<.001). Participants lost to follow-up interacted a mean of 6.0 (SD 8.0) times with the program. In comparison, those who were assessed interacted a mean of 28.2 (SD 14.2) times with the program. This means that 64.8% (276/426) of the students who interacted 0-30 times with the program were assessed at follow-up in comparison with 98.4% (184/187) of those with 31-40 interactions, and 100% (137/137) of those with 41-50 interactions. The original study underlined the appropriateness of the program for the target group with most of its participants (710/750, 94.7%) staying subscribed until month 6. In addition, a large proportion of the participants stated that they read the SMS text messages (551/563, 98.4%, with valid follow-up data) and rated the program as helpful (384/550, 69.8%) and individually tailored (327/550, 59.5%). Intention-to-treat comparisons at the 6-month follow-up illustrated significant differences between both groups in terms of reducing the number of drinks consumed per month (intervention group vs control group: –0.6 vs 0.7; *P*=.03) and the number of cigarettes smoked per month (intervention group vs control group: –1.7 vs 5.0; *P*=.01), as well as reported stress (*P*=.02). These are initial results because the analyses of the 18-month follow-up data are ongoing.

The intervention was designed with, and initiated by, the open-source behavioral intervention platform *MobileCoach* [38,39]. The original study protocol was approved by the ethics committee of the Faculty of Arts and Sciences at the University of Zurich, Switzerland (approval number 18.6.5; date of approval: June 21, 2018). The study was registered with ISRCTN (ISRCTN41347061; assigned July 21, 2018) and executed in full compliance with the Declaration of Helsinki.

### Description of SmartCoach

**Figure 1:**
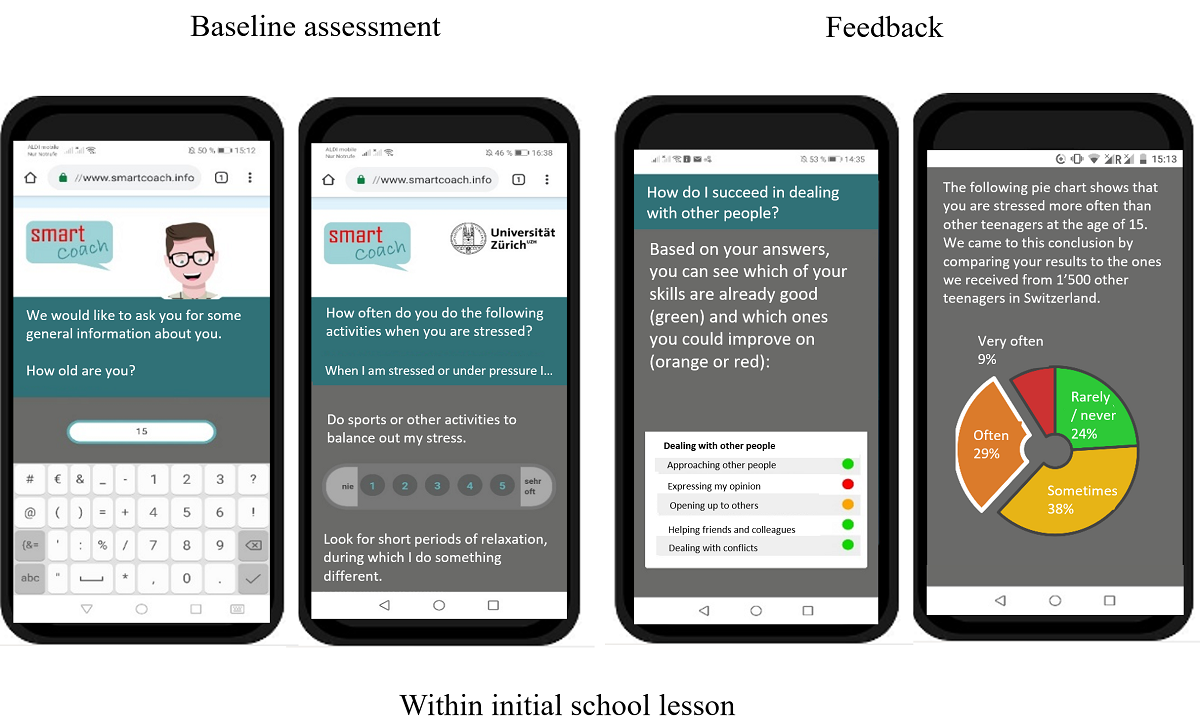
Overview of the *SmartCoach* survey components.

**Figure 2:**
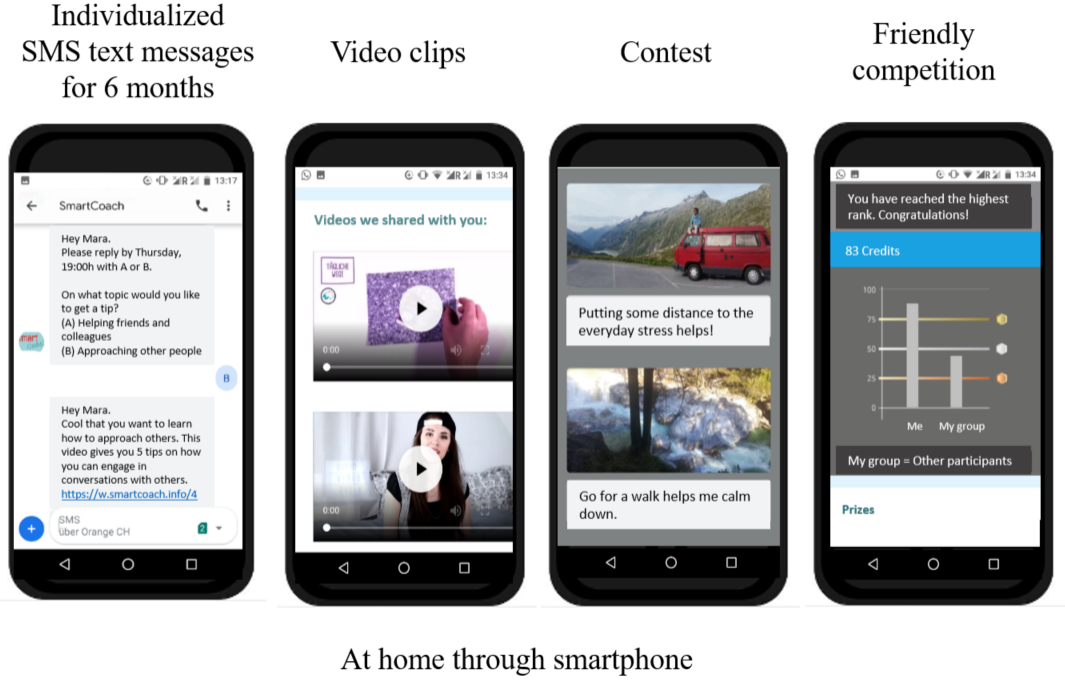
Overview of the *SmartCoach* intervention components.

Figures 1 and 2 depict all components of the *SmartCoach* program. The *SmartCoach* program combined (1) tailored web-based feedback to reduce the individual level of stress, which was delivered directly after completion of the baseline assessment, and (2) tailored mobile phone SMS text messages to promote self-management skills (block 1), social skills (block 2), and substance use resistance skills (block 3) over 6 months. The theoretical backgrounds of these intervention components are described elsewhere [14,18].

During the 6-month *SmartCoach* program, participants received one SMS text message prompt per week that (1) introduced 1 of the 3 life skills blocks, (2) or encouraged the subject’s participation in quizzes, self-challenges, and individual stress or social skills trainings, or (3) invited participants to post pictures or messages and vote on other posts published by all intervention participants at the end of a block. The initial SMS text messages included graphical objects but did not require an answer. The prompts encouraging the subject’s participation in quizzes, self-challenges, and individual stress or social skills trainings were easily answered by typing a single letter or number using the mobile phone’s reply function. Answering such a prompt could trigger 1-2 further SMS text messages in the program. Some of these answers included hyperlinks to thematically relevant video clips, pictures, and related websites. To participate in the contests, the participants had to upload a picture or post a motivational text message on a website within 2 days. This was followed by a 2-day window for voting on all posts and a presentation of the 3 contributions with the highest votes on day 5. This function was also available on the website. Table 1 displays the sequence and contents of the SMS text messages and the possible activities prompted by these SMS text messages.

**Table 1:** Sequence, content, and engagement with different prompts within the *SmartCoach* program (N=750).

Week and content	Required activities	Students engaging with activity, n (%)
**1: Introduction to self-management skills and quiz on origin and function of stress**		
	Click on link to an overview picture	194 (25.9)
	Reply to quiz question	525 (70)
	Click on video link	444 (59.2)
**2: Quiz on common stressors**		
	Reply to quiz question	513 (68.4)
	Click on video link	410 (54.7)
**3 and 6: Tailored stress reduction strategies for individual stressors**		
	Reply to SMS text message with options	938 (62.5)^a^
	Click on video or website link	605 (40.3)^a^
**4: Self-challenge on general stress reduction strategies**		
	Reply to SMS text message with options	465 (62)
	Reply to SMS text message on successful application of chosen strategy	471 (62.8)
**5: Quiz on eustress vs distress**		
	Reply to quiz question	473 (63.1)
	Click on video link	359 (47.9)
**7: Group contest on preferred stress management strategy**		
	Viewing of others’ posts	480 (64)^b^
	Posting a picture and message on individually preferred strategy	193 (25.7)
	Voting on others’ posts	222 (29.6)
**8: Introduction to social skills and quiz on social skills**		
	Click on link to an overview picture	372 (49.6)
	Reply to quiz question	416 (55.5)
	Click on link to picture	301 (40.1)
**9: Tailored strategies for improving personal social skills**		
	Reply to SMS text message with options	716 (95.5)
	Click on video or website link	398 (53.1)
**10: Quiz on use of body language in different situations**		
	Reply to quiz question	426 (56.8)
	Click on video link	299 (39.9)^c^
**11: Tailored strategies for improving personal social skills**		
	Reply to SMS text message with options	417 (55.6)
	Click on video or website link	231 (30.9)
**12: Self-challenge on strategies to improve social skills in different areas**		
	Reply to SMS text message with options	403 (53.7)
	Reply to SMS text message on successful application of chosen strategy	416 (55.5)
**13: Origin of smartphone addiction**		
	Reply to quiz question	418 (55.7)
	Click on video link	294 (39.2)
**14: Quiz on associations between smartphone use and stress tailored to gender**		
	Reply to quiz question	384 (51.2)
	Click on video links	360 (48)
**15: Self-challenge on smartphone detox**		
	Reply to SMS text message with options	386 (51.5)
	Reply to SMS text message on successful detox in chosen situation	405 (54)
	Click on video link	239 (31.9)
**16: Quiz on recognition of peer pressure**		
	Click on link to the first part of the video	368 (49.1)
	Reply to quiz question	354 (47.2)
	Click on link to the second part of the video	373 (49.7)
**17: Group contest on favourite social situation**		
	Viewing of others’ posts	393 (52.4)^b^
	Posting a picture and message on favourite social situation	91 (12.1)
	Voting on others’ posts	144 (19.2)
**18: Introduction to substance use resistance skills and quiz on substance use prevalence (alcohol or tobacco) in reference group and normative feedback**		
	Click on link to an overview picture	312 (41.6)
	Reply to quiz question	387 (51.6)
**19: Quiz on the presence of tobacco advertisements directed to adolescents in everyday life**		
	Reply to quiz question	369 (49.2)
	Click on video link	246 (32.8)
**20: Quiz on risks of alcohol use**		
	Reply to quiz question	386 (51.5)
	Click on website link	238 (31.7)
**21: Tailored information on social consequences of alcohol use**		
	Click on video link	323 (43.1)
**22: Group contest on motivation for abstinence or low-risk alcohol use**		
	Viewing of others’ posts	393 (52.4)^b^
	Posting a motivational SMS text message	106 (14.1)
	Voting on others’ posts	132 (17.6)

^a^The MobileCoach log files do not include a timestamp for this activity. The numbers display the total engagement with this task in the corresponding week 3 and week 6.

^b^The MobileCoach log files do not include a timestamp for this activity. The numbers display the total engagement with this task in the corresponding week.

^c^Information is missing for 1 media object (out of 4) included within the SMS text message replies. This number might underestimate the total engagement of the students with this task.

### Measures

After providing informed consent, the students participated in a baseline assessment during a regular class session through which data on potential predictors of engagement and outcome variables were collected. The sociodemographic characteristics that were assessed were gender, age, immigrant background, and type of school (secondary or upper secondary school). We assessed the country of birth of both parents of the students to identify any potential immigrant background. On the basis of this information, the participants were assigned to one of the following categories: (1) neither parent born outside Switzerland, (2) 1 parent born outside Switzerland, or (3) both parents born outside Switzerland.

The health-related variables that were assessed were physical activity, well-being, perceived stress, social skills, problem drinking, tobacco smoking, and cannabis use.

Self-reported moderate to vigorous physical activity was measured using a question derived from the Health Behaviour in School-aged Children study [40]: “Outside school, how many hours a week do you exercise or participate in sports that make you sweat or out of breath?” Well-being was assessed using the World Health Organization–5 Well-being Index [41]. Perceived stress was measured using a single item from the Swiss Juvenir study [42]: “How often have you had the feeling of being overstressed or overwhelmed in the last month?” Participants were asked to indicate their response on a 5-point Likert scale that ranged from *never* to *all the time*. Social skills were assessed by using the brief version of the Interpersonal Competence Questionnaire [43]. Problem drinking was assessed by using the Alcohol Use Disorders Identification Test–Concise (32) with a cut-off of ≥5 based on a large German sample [44]. Tobacco smoking was assessed following the criteria of the Society for Nicotine and Tobacco Research [45] and using the question “Have you smoked a puff within the last 30 days?” Cannabis use was assessed by an item of the Health Behaviour in School-aged Children study [46] addressing the number of cannabis consumption days in the last 30 days.

Use of the program was assessed in terms of the total number of interactions; the number of responses to the weekly SMS text message prompts (quizzes, self-challenges, and individual stress and skills trainings); the number of retrieved media objects within the program (videos, pictures, and website links); and the number of views, posts, and votes within contests. This information was available through the log files of the MobileCoach system.

The subjective experience was assessed at 6-month follow-up by asking the students to report on how attentively they had read the SMS text messages, with the possible answers *thoroughly*, *quickly,* and *not at all*. The answers *quickly* and *not at all* were merged into 1 group.

### Statistical Analyses

Descriptive statistics were used to examine participant use of the *SmartCoach* program. Bivariate correlations were performed to evaluate relationships among the use variables. The use variables correlated with each other with scores from *r*=0.63 (moderate) and *r*=0.90 (high). For further analysis, the following use outcomes were selected:

1. Total number of interactions with the program, referred to as overall engagement and encompassing all components of the intervention.
2. Total number of quizzes answered.
3. Total number of media objects retrieved.
4. Total number of interactions within contests.

The rationale for selecting the variables 2, 3, and 4 was based on the stipulated effort made by the participant from low (answering a quiz question by typing a predefined letter) to middle (downloading and watching a video) to high (thinking about their own post or voting on other posts within a contest). All use variables were evaluated for normality using Q-Q plots and Shapiro–Wilk tests. According to these checks, all variables demonstrated nonnormal distributions. Finally, associations between overall use and subjective experience were examined.

For detecting relevant individual characteristics associated with engagement (use and subjective experience) with the program, a conditional inference tree, referred to throughout the paper also as decision tree, was estimated [47]. In short, a decision tree follows these steps iteratively:

1. First, it tests for all input variables for which the variable is independent from the response variable (=null hypothesis). If this null hypothesis is not rejected for any input variable, the decision tree stops. Otherwise, it selects the input variable that shows the strongest dependency.
2. On the basis of the selected input variable, it splits the sample into 2 groups.
3. It repeats steps 1 and 2 in both groups until the process stops or until the groups become too small [48].

The advantages of decision trees are as follows: (1) outcomes and residuals do not need to meet assumptions about their distribution, (2) the findings are easy to interpret, and (3) larger amounts of predictors as well as their interactions can be tested simultaneously [47]. The main disadvantages of decision trees are as follows: (1) variable selection is affected by order effects known also from other stepwise variable selection approaches applied within regression analysis, and (2) the strong dependency of the chosen predictors and cut points from the distribution of the observations in the given sample.

Thus, to assess the stability of decision trees, a toolkit of graphical illustrations was used based on resampling [49]. The basic idea of this method is the following: it repeatedly draws new, artificial data sets—so-called bootstrap samples—from the original data set and constructs a decision tree in the new data set. Each bootstrap sample is regarded as a plausible outcome if the study were to be repeated in a new sample. If a decision tree in the bootstrap sample has a similar structure and also selects a similar set of predictor variables, this is interpreted as an indication that these variables have a stable relationship with the outcome variable. In this study, 500 bootstrap samples were drawn for each of the engagement outcomes, and it was investigated whether the predictors that were selected in the original trees were also consistently selected for splitting in the resampled data sets and how often (on average) they were selected for prediction.

In a last step, associations among the engagement outcomes, as mentioned previously, and pre–post changes in the primary and secondary outcomes of the original study were examined. For binary outcomes, follow-up values were included as dependent variables and baseline values, and engagement outcomes (one at a time) were included as independent variables. For continuous outcomes, differences from baseline to 6-month follow-up were included as dependent variables, whereas independent variables were the same as for binary outcomes.

R software (version 3.6.3; The R Foundation for Statistical Computing) and the packages partykit [50,51] and stablelearner [49] were used to perform recursive partitioning and stability assessment, whereas SPSS software (version 22; IBM Corp) was used for all other analyses. All statistical tests were 2-tailed, with *P*<.05 set as the criterion for statistical significance.

## Results

### Participants

Table 2 presents baseline characteristics that were used to predict engagement. Of the 750 participants analyzed for this study, 423 (56.4%) were girls. The reported mean age was 15.4 (SD 1.0) years. Almost half of the participants (361/750, 48.2%) reported either a 1-sided or 2-sided immigrant background. Three-fourth (585/750, 78%) of the participants were recruited at an upper secondary school, and the remaining one-fourth (165/750, 22%) were recruited at a secondary school. As expected for this cohort of younger students, only some of the participants reported problem drinking (114/750, 15.2%) and tobacco (91/750, 12.1%) or cannabis use (106/750, 14.1%) 30 days before baseline assessment. The participants reported feeling rather stressed before baseline (mean 2.9, SD 0.9; Q1, Q3=2, 4). In addition, the mean Well-being Index score was 52.9 (SD 17.3; Q1, Q3=40, 76) out of 100 possible points. On the basis of the Interpersonal Competence Questionnaire, brief form, the mean level of interpersonal competences was rather high.

**Table 2:** Baseline characteristics of the study sample (N=750).

Variable			Values
**Sex, n (%)**			
	Male	327 (43.6)	
	Female	423 (56.4)	
Age (years), mean (SD)			15.4 (1.0)
Number of physically active hours per week^a^, mean (SD)			4.1 (3.5)
**Immigration background, n (%)**			
	No immigration background	389 (51.9)	
	One parent born outside Switzerland	173 (23.1)	
	Both parents born outside Switzerland	188 (25.1)	
**Type of school, n (%)**			
	Secondary school	165 (22)	
	Upper secondary school	585 (78)	
Tobacco smoking before baseline, yes, n (%)			91 (12.1)
Problem drinking before baseline, yes, n (%)			114 (15.2)
Cannabis use before baseline, yes, n (%)			106 (14.1)
Perceived stress on a scale of 1-5, mean (SD)			2.9 (0.9)
Well-being (WHO^b^-5 Well-being Index) on a scale of 1-100, mean (SD)			52.9 (17.3)
Interpersonal competences (ICQ-10^c^) on a scale of 5-20, mean (SD)			14.9 (2.2)

^a^Missing values: n=1.

^b^WHO: World Health Organization.

^c^ICQ-10: Interpersonal Competence Questionnaire, brief form.

### Use of Different Program Components

Table 3 summarizes different program use characteristics across the sample. Of the 750 students, 40 (5.3%) discontinued the intervention actively by sending an SMS text message to the program. Of these 40 students, 17 (43%) signed off at program start, whereas the rest (23/40, 58%) signed off somewhere between the first and 16th program weeks.

The participants replied to an average of 23.6 (SD 15.9) of 50 program prompts. They interacted with the program mainly through SMS text message and the least by participating in a picture or message contest involving all intervention participants. They answered a mean of 12 (SD 7.7) of 21 SMS text message prompts. On average, the participants downloaded 8.8 (SD 6.9) of 20 media objects received. In all, 3 contests were prompted throughout the program, each of them including 3 steps (viewing, posting, and voting). Although 50% (375/750) of the participants viewed at least two contests, only a few participants accepted the invitation to post pictures or messages or voted on them within the contests. No participant posted something during all 3 contests.

Table 1 displays the number of students engaging with the prompted activities of the program each week. In general, engagement with the program decreased over time, as can be observed for the most popular activity, replying to quiz questions (week 1=525/750, 70%; week 20=386/750, 51.5%). However, the trend is not linear, and there are weeks with engagement peaks depending on the topic (eg, in week 8, 372/750, 49.6%, clicked on the link to an overview of the social skills topic, whereas only 194/750, 25.9%, did so in week 1; in week 9, 716/750, 95.5%, replied to the SMS text messages with options for improving their social skills).

**Table 3:** Use of program components.

Engagement variables		Values, mean (SD)	Values, median (Q1^a^, Q3^b^)	Range
Total number of interactions		23.6 (15.9)	26.5 (8, 38)	0-50
**SMS text messages**				
	Quizzes	6.2 (4.0)	7 (2, 7)	0-11
	Stress-trainings	1.3 (0.9)	2 (0, 2)	0-2
	Self-challenges	3.4 (2.4)	4 (1, 6)	0-6
	Skill-trainings	1.1 (0.9)	1 (0, 2)	0-2
	Total use of text messages	12.0 (7.7)	14 (4, 19)	0-21
**Media objects**				
	Videos	5.1 (4.0)	5 (1, 9)	0-12
	Pictures	1.9 (1.5)	2 (1, 3)	0-4
	Website links	1.7 (1.7)	1 (0, 3)	0-6
	Total use of media objects	8.8 (6.9)	9 (2, 15)	0-20
**Picture and SMS text message contests**				
	Views	1.8 (1.3)	2 (0, 3)	0-3
	Posts	0.3 (0.4)	0 (0, 1)	0-1
	Votes	0.7 (0.9)	0 (0, 1)	0-3
	Total use of contests	2.9 (2.5)	3 (0, 5)	0-9

^a^Q1: quartile 1.

^b^Q3: quartile 3.

### Predictors of Engagement

#### Predictors of Total Use

**Figure 3:**
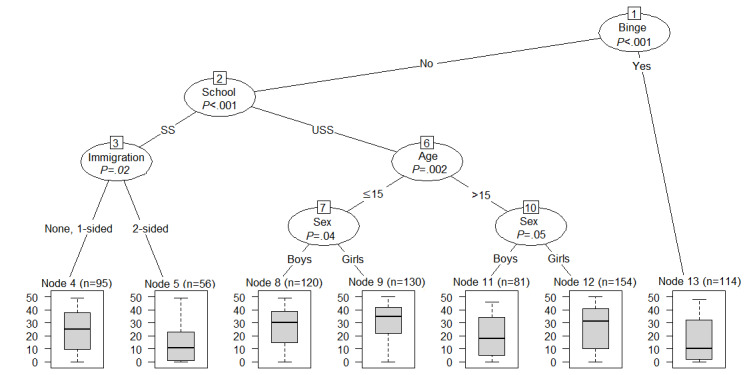
Decision tree with predictors of overall use of the *SmartCoach* program. Binge: problem drinking; Immigration: immigration background; School: school type; SS: secondary school; USS: upper secondary school.

Figure 3 depicts a decision tree for the prediction of overall use of the *SmartCoach* program. Baseline characteristics used for prediction of overall use were problem drinking before baseline, school type, immigration background, age, and sex (Figure 3). The decision tree can be read as follows: students who reported problem drinking before baseline (node 13) and students who did not drink in a problematic manner before baseline but attended a secondary school and reported a 2-sided immigration background (node 5) were expected to show low use of the program. The highest use was expected for girls aged ≤15 years who attended an upper secondary school and who reported no alcohol use or moderate alcohol use before baseline (node 9). Girls at upper secondary schools used the program more than boys at upper secondary schools. At secondary schools, use of the program was similar across both sexes.

Multimedia Appendix 1 gives an insight into the stability of this decision tree. Each column indicates how often each predictor variable was selected in the 500 bootstrap samples. Whereas variables selected in the original tree are presented as black columns, variables that were not selected in the original tree are presented as white columns. The predictors problem drinking and school type were selected very often (approximately 80% of the time) for the prediction of overall use and therefore showed a relatively stable relationship with the outcome. However, the predictors sex, age, and immigration background were only represented in less than half of the trees generated based on the bootstrap samples, which makes them less reliable for prediction compared with the predictors problem drinking and school type. Tobacco smoking, cannabis use, perceived stress, and interpersonal competences were not represented in the original tree but appeared in 10%-15% of the other decision trees. This indicates that although those variables seem to carry some information that could be useful for predicting overall use, they are not predominant.

#### Predictors of Quiz Use

**Figure 4:**
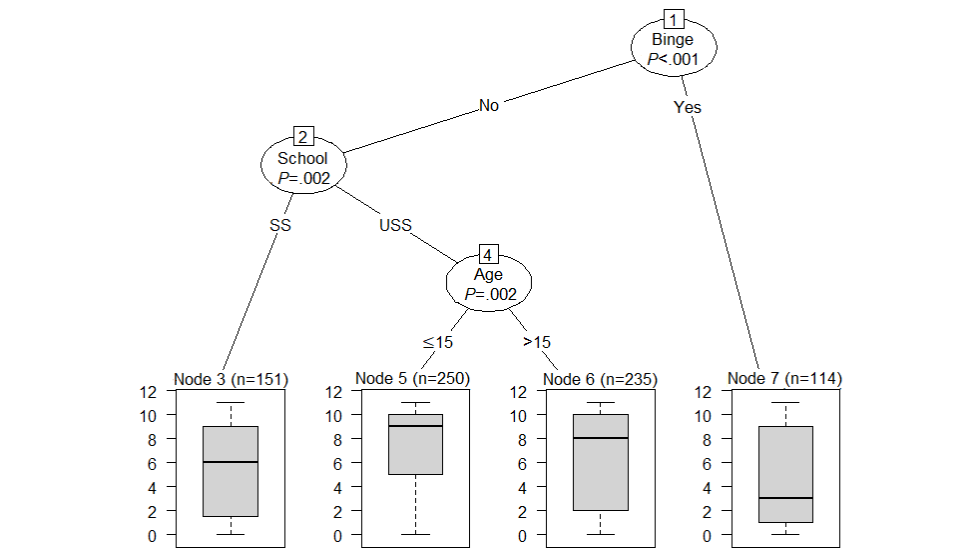
Decision tree for the prediction of use of quizzes. Binge: problem drinking; School: school type; SS: secondary school; USS: upper secondary school.

Figure 4 depicts a decision tree for quiz use, encompassing the following predictors: problem drinking, school type, and age. However, the stability check (Multimedia Appendix 2) implies that only problem drinking showed a stable relationship with the outcome, being chosen in 80% (400/500) of the subsamples for prediction. School type was chosen only in approximately 50% of the 500 decision trees and age in less than 50% and almost as often as immigration background, which was not depicted in the original tree. To sum up, this decision tree should be read as follows: nondrinkers at baseline showed higher use of quizzes, whereas students who reported problem drinking at baseline showed lower use.

#### Predictors of Media Use

**Figure 5:**
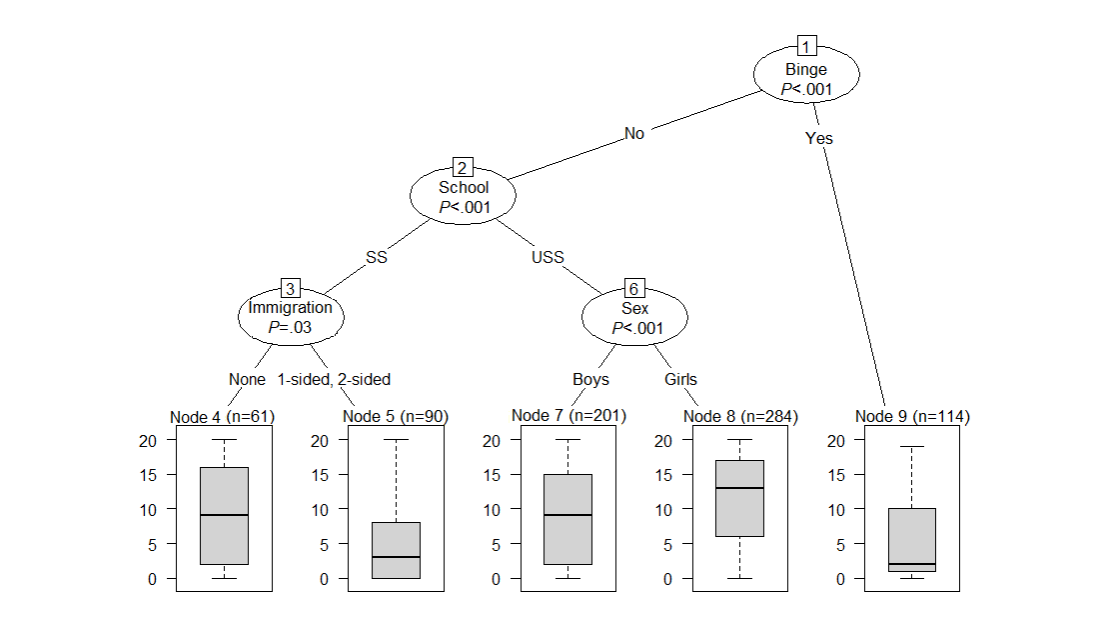
Decision tree for the prediction of use of media objects. Binge: problem drinking; Immigration: immigration background; School: school type; SS: secondary school; USS: upper secondary school.

Figure 5 illustrates a decision tree for use of media objects, encompassing the following predictors: problem drinking, school type, sex, and immigration background. However, the stability check (Multimedia Appendix 3) implies that immigration background has a less stable relationship with the use of media objects because it was only chosen in approximately 30% of the decision trees and almost as often as tobacco smoking, age, and cannabis use. The other predictor variables showed a more stable relationship. This once again could be read as follows: students who reported problem drinking at baseline were expected to use media objects the least, whereas regular use was expected across nondrinkers assisting different school types with the only difference being that girls at upper secondary schools used this program component more than the rest.

#### Predictors of Contest Use

**Figure 6:**
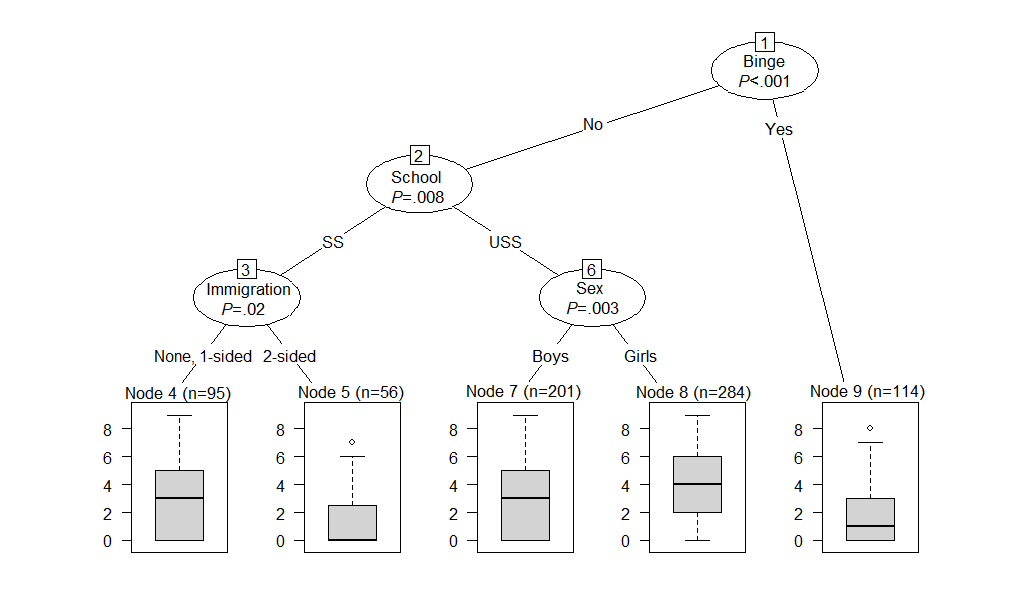
Decision tree for the prediction of use of contests. Binge: problem drinking; Immigration: immigration background; School: school type; SS: secondary school; USS: upper secondary school.

Figure 6 depicts a decision tree for use of contests, encompassing the following predictors: problem drinking, school type, sex, and immigration background. The tree looks very similar to the one for media objects, with the only difference being a split in the immigration background. However, the stability check (Multimedia Appendix 4) implies that none of these predictors showed a highly stable relationship with the use of contests because they only appear approximately 50% of the time or even less. This decision tree should therefore not be considered for further interpretation.

#### Predictors of Subjective Experience

**Figure 7:**
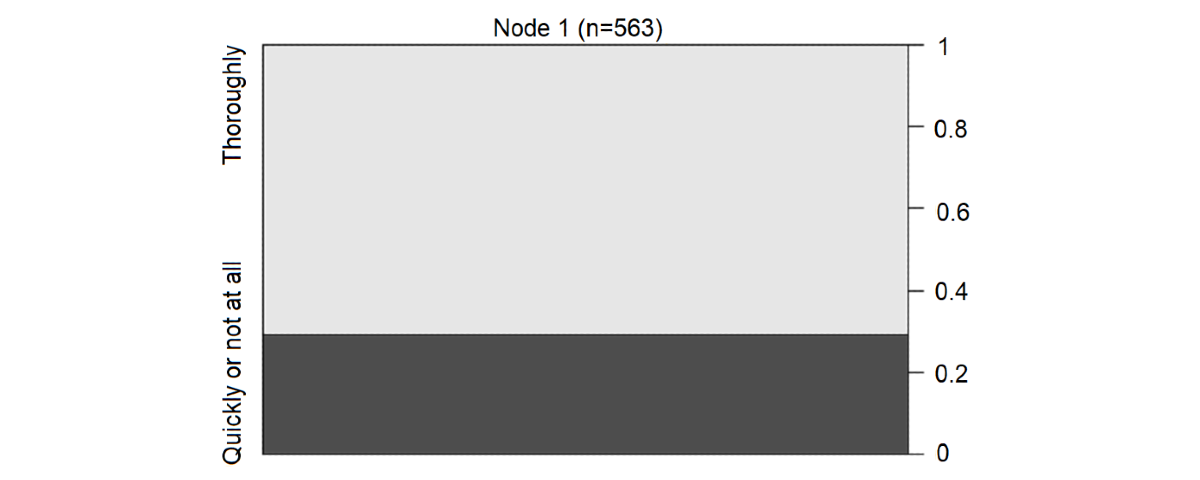
Decision tree for the prediction of subjective experience, which has no splits and consists of a single node.

Figure 7 depicts the result of the decision tree for predicting the subjective experience of students with the program. As it becomes clear from Figure 7 and from Multimedia Appendix 5, none of the included predictors showed a stable relationship with the outcome.

### Associations Among Use, Subjective Experience, and Changes in Outcomes

Overall use of the program was not predictive in our complete case analysis for problem drinking (odds ratio [OR] 0.99, 95% CI 0.03-0.09; *P*=.65) or cannabis smoking (OR 0.98, 95% CI 0.97-1.01; *P*=.20) at follow-up or for changes in interpersonal competences (*βs*=.2; t_571_=0.41; *P*=.68).

A tendency was observed for the outcome tobacco use, where participants who interacted more frequently with the program showed lower odds of having smoked at follow-up (OR 0.98, 95% CI 0.96-1.00; *P*=.05; *R^2^*=0.18) compared with baseline. Ancillary analysis on tobacco smoking revealed that use of contests compared with the rest of the program components lowered the odds of having smoked at follow-up (OR 0.86, 95% CI 0.76-0.98; *P*=.02; *R^2^*=0.18). Finally, a significant quadratic association was established between overall use (*βs*=.39; t_586_=2.66; *P*=.008; *R^2^*=0.24) of the program and pre–post changes in well-being assessed using the World Health Organization–5 Well-being Index. All participants showed an increase in well-being from baseline to 6-month follow-up, but the mean increase was greater for lower- and higher-engaged participants. The highest increase in well-being was observed for participants who participated in almost all program prompts.

Subjective experience (assessed by asking the students to report on how attentively they had read the SMS text messages) was predictive for problem drinking at follow-up. Students who read the SMS text messages more attentively were significantly less at risk for problem drinking at follow-up (OR 0.43, 95% CI 1.29-3.41; *P*=.003; *R^2^*=0.19).

## Discussion

### Principal Findings

Using a proactively recruited sample of young adolescents in secondary and upper secondary schools, this study examined (1) the use of, and subjective experience with, a digital life skills intervention; (2) the associations between program engagement and adolescent characteristics; (3) the stability of these associations using a resampling method; and (4) the associations between program engagement and changes in health-related outcomes.

The main findings are as follows:

1. Adolescents took part in half of the interactions prompted by the program, with SMS text messages being the most used and contests being the least used components.
2. Of all adolescent characteristics included in the decision tree analysis, the following were chosen most often as a predictor of engagement: problem drinking, school type, age, sex, and immigration background. In almost all decision trees, adolescents who reported problematic patterns of alcohol use at baseline were expected to use the program the least, followed by adolescents attending a secondary school and reporting a 2-sided immigration background. Often, a higher use of the program and its different components was expected for girls and younger adolescents (aged ≤15 years).
3. Predictors included in the original trees were often considered unstable for prediction after resampling. The most stable predictor for overall and component use after resampling was problem drinking before baseline (relative frequency [RF] of approximately 80% in 3 out of 4 trees), followed by school type (RF≥80% in 2 out of 4 trees). Predictors of contest use and subjective experience were especially unstable (RF≤50%).
4. Adolescents who used the contests more intensively were more likely to be nonsmokers at follow-up compared with those who did not. In addition, participants who interacted the most and the least with the program were more likely to increase their well-being from baseline to 6-month follow-up. Finally, adolescents who read the SMS text messages more attentively were less likely to drink in a problematic manner at follow-up.

This is the first study to examine engagement with a mobile phone–based life skills intervention among younger adolescents. Similar results were found for 2 other mobile phone–based interventions directed at older adolescents, namely that SMS text message prompts and especially quizzes were the most used, whereas contests, where a message has to be produced by the participant, were the least used [12,29]. However, this study showed that contest use was associated with the probability of being a nonsmoker at follow-up, and a recent review on digital mental health interventions [21] concluded that program components enabling interaction with peers were the most engaging, which is why this component should not be eliminated from the intervention without further examination.

The results of this study coincide with previous research, identifying younger adolescents [27,29], women [21], and adolescents without an immigration background [29] to engage more with digital interventions aimed at promoting mental health or preventing substance use. However, this study is the first to not only examine predictors of engagement, but to also analyze the stability of these predictors, and in doing so, to overcome order effects in variable selection and dependency from the recruited sample known from standard regression analysis. As a result, sex, age, and immigration background were detected as rather unstable predictors, which is why associations between these adolescent characteristics and engagement outcomes should be considered only with caution.

The most stable predictor over all decision trees was problem drinking reported at baseline. Generally speaking, adolescents who already showed patterns of problematic alcohol use at baseline were expected to engage the least with the program. The same association could not be found for tobacco or cannabis use, probably because the included items measured prevalence instead of severity of use, but this remains a question for future research. In other words, for those adolescents who are already involved in risky substance use, an unspecific intervention might not be appealing because they cannot infer their benefit. This would coincide with previous research, which found a tobacco-specific intervention to be more engaging for adolescents with higher self-perceived benefit in quitting smoking [29] and a mental health intervention to be more engaging for adolescents with higher levels of depressive symptoms [27]. Future qualitative work is needed to ascertain if digital life skills interventions should be improved for adolescents who are already involved in risky substance use or if a problem-specific digital intervention could be of more interest for this subgroup of adolescents.

Another rather stable predictor was school type, which can be interpreted as a proxy for educational level in this study. The results indicate that the mobile phone–based life skills intervention was more engaging for upper secondary school students than for secondary school students. This suggests that the intervention could have been still too demanding for students with lower educational attainment. Specifically, media objects—but not quizzes—could have been too demanding for those adolescents. However, interaction with quizzes alone was not found to be associated with changes in health-related outcomes, indicating that despite being engaging for users, quizzes are less useful for fostering behavior change and more useful for ensuring program use and for preventing program dropout. New ways to make media objects more engaging, including for those with lower educational attainment, must be explored. A novel chatbot-based life skills intervention that was developed in a participatory manner with 20 adolescents [16] showed that simple cartoon videos were more appealing than text information and more useful for fostering self-reflection on individual life skills if they were presented at the beginning of a session. Cooperating with organizations that already produce engaging and relevant digital content for adolescents, as planned by the developers of a novel mobile-based intervention to support social emotional learning and identity development in adolescents from low-resource contexts [17], could also increase the use of such components within digital life skills interventions.

The results of this study suggest that there is a positive association between engagement and intended outcomes. A higher use of contests and the overall program were associated with intended tobacco use and well-being outcomes. In addition, reading the text messages more attentively was associated with intended alcohol use at follow-up. However, low use of the program also predicted higher well-being at follow-up compared with baseline. These mixed results could stem from the different reach at follow-up of more-engaged compared with less-engaged participants, which is a rather well-known problem from previous studies [29,52] or from the fact that less-engaged participants received sufficient support to facilitate intrinsic motivation to boost well-being [53]. Both explanations are plausible when looking into previous studies with adults, where less-engaged participants with access to mobile phone–based smoking reduction programs reported greater changes at follow-up [54,55]. Challenges for future research remain finding ways to also assess lower-engaged participants at follow-up. The strategy of this study—reimbursement of CHF 10 (US $10.90) for participation at each follow-up and up to 5 follow-up calls—seems to be insufficient. A possible way to combat this problem for studies recruiting in school settings could be to conduct follow-up assessments within regular school sessions.

### Limitations

The findings of this study must be interpreted in view of its limitations. First, answers to weekly prompts were rewarded with credits, and credits were linked to a prize draw. Second, as already emphasized in other studies [29,55], the quantity and quality of answers to prompts could differ (eg, an adolescent who answers all quiz questions but answers them all wrong). Rather than just analyzing registered events, future qualitative work should investigate whether the content of answers or contest posts is associated with treatment outcomes. Furthermore, the results of this study rely on a convenience sample, and the findings might not be generalizable to the entire population. Finally, this study relied on self-report data, which bears the risk that the results may have been influenced by social desirability.

### Conclusions

In summary, in our study, adolescents who did not drink in a problematic manner before program start frequently engaged with a mobile phone–based life skills intervention, regardless of their sex, age, and immigration background. Further efforts should be undertaken to reach adolescents through digital life skills interventions before they become involved with risky substance use. Digitally delivered life skills interventions must carefully consider how the proportion between engaging and change-modeling components should be weighed to comply equally with equity standards and intended intervention outcomes. In addition, future studies could go a step further and make assumptions about *effective* engagement but only in combination with strategies that are able to reduce attrition bias at follow-up.

## Appendix

Multimedia Appendix 1 Corresponding assessment for predictor stability of the decision tree for the prediction of overall use. Binge: problem drinking; Cannabis: cannabis use; ICQ: Interpersonal Competence Questionnaire score; Immigration: immigration background; Physical: physical activity; School: school type; Stress: perceived stress; Tobacco: tobacco use; WHO: World Health Organization–5 Well-being Index score.

Multimedia Appendix 2 Corresponding assessment for predictor stability of the decision tree for the prediction of use of quizzes. Binge: problem drinking; Cannabis: cannabis use; ICQ: Interpersonal Competence Questionnaire score; Immigration: immigration background; Physical: physical activity; School: school type; Stress: perceived stress; Tobacco: tobacco use; WHO: World Health Organization–5 Well-being Index score.

Multimedia Appendix 3 Corresponding assessment for predictor stability of the decision tree for the prediction of use of media objects. Binge: problem drinking; Cannabis: cannabis use; ICQ: Interpersonal Competence Questionnaire score; Immigration: immigration background; Physical: physical activity; School: school type; Stress: perceived stress; Tobacco: tobacco use; WHO: World Health Organization–5 Well-being Index score.

Multimedia Appendix 4 Corresponding assessment for predictor stability of the decision tree for the prediction of use of contests. Binge: problem drinking; Cannabis: cannabis use; ICQ: Interpersonal Competence Questionnaire score; Immigration: immigration background; Physical: physical activity; School: school type; Stress: perceived stress; Tobacco: tobacco use; WHO: World Health Organization–5 Well-being Index score.

Multimedia Appendix 5 Corresponding assessment for predictor stability of the decision tree for the prediction of subjective experience. Binge: problem drinking; Cannabis: cannabis use; ICQ: Interpersonal Competence Questionnaire score; Immigration: immigration background; Physical: physical activity; School: school type; Stress: perceived stress; Tobacco: tobacco use; WHO: World Health Organization–5 Well-being Index score.

## Abbreviations

OR: odds ratio
RF: relative frequency
