# Supplementary figures and images for: Engagement With a Mobile Phone–Based Life Skills Intervention for Adolescents and Its Association With Participant Characteristics and Outcomes: Tree-Based Analysis
Source: J Med Internet Res. 2022 Jan 19;24(1):e28638. doi: 10.2196/28638 (PMC8811696; doi:10.2196/28638)

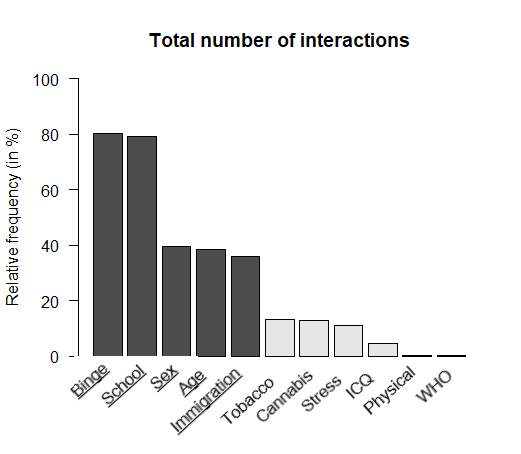

Supplement: Multimedia Appendix 1 [file jmir_v24i1e28638_app1.png]

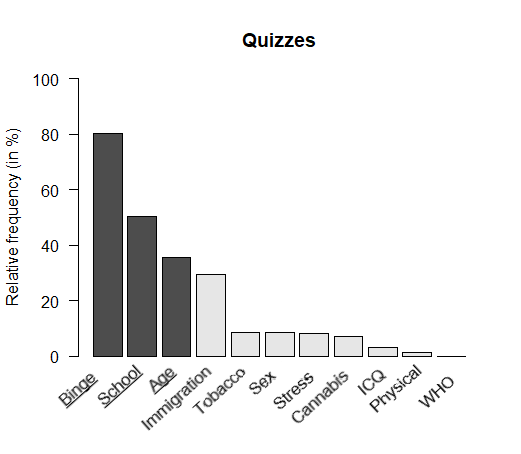

Supplement: Multimedia Appendix 2 [file jmir_v24i1e28638_app2.png]

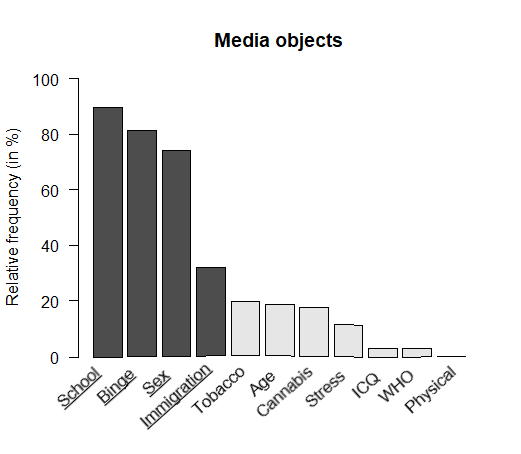

Supplement: Multimedia Appendix 3 [file jmir_v24i1e28638_app3.png]

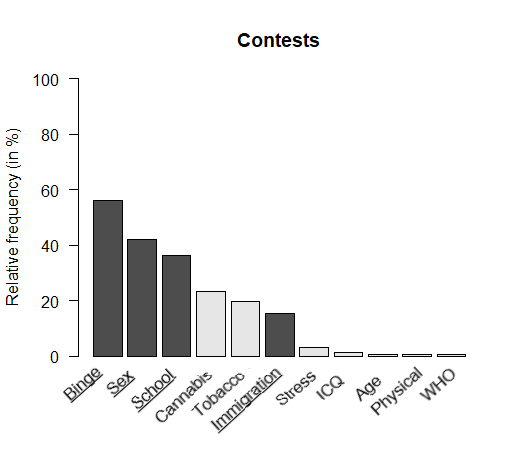

Supplement: Multimedia Appendix 4 [file jmir_v24i1e28638_app4.png]

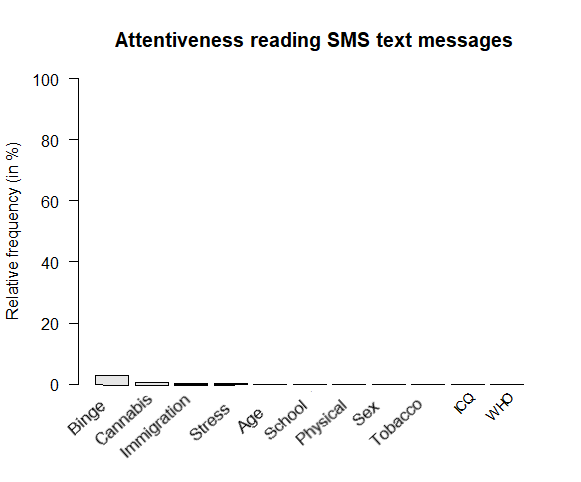

Supplement: Multimedia Appendix 5 [file jmir_v24i1e28638_app5.png]
